# The Emerging Role of Plant-Derived Exosomes-Like Nanoparticles in Immune Regulation and Periodontitis Treatment

**DOI:** 10.3389/fimmu.2022.896745

**Published:** 2022-06-10

**Authors:** Zeyu Zhang, Yang Yu, Guanxiong Zhu, Liting Zeng, Shaofen Xu, Haoyu Cheng, Zhaoguang Ouyang, Jianwei Chen, Janak L. Pathak, Lihong Wu, Lina Yu

**Affiliations:** ^1^ Department of Preventive Dentistry, Affiliated Stomatology Hospital of Guangzhou Medical University, Guangdong Engineering Research Center of Oral Restoration and Reconstruction, Guangzhou Key Laboratory of Basic and Applied Research of Oral Regenerative Medicine, Guangzhou, China; ^2^ School and Hospital of Stomatology, Guangzhou Medical University, Guangzhou, China; ^3^ Department of Sports and Health, Guangzhou Sport University, Guangzhou, China

**Keywords:** plant-derived exosome-like nanoparticles, periodontitis, exosomes, inflammation, oral tissue regeneration, drug delivery systems, extracellular vesicles

## Abstract

Periodontitis is an infectious oral disease, which leads to the destruction of periodontal tissues and tooth loss. Although the treatment of periodontitis has improved recently, the effective treatment of periodontitis and the periodontitis-affected periodontal tissues is still a challenge. Therefore, it is urgent to explore new therapeutic strategies for periodontitis. Natural products show anti-microbial, anti-inflammatory, anti-oxidant and bone protective effects to periodontitis and most of these natural products are safe and cost-effective. Among these, the plant-derived exosome-like nanoparticles (PELNs), a type of natural nanocarriers repleted with lipids, proteins, RNAs, and other active molecules, show the ability to enter mammalian cells and regulate cellular activities. Reports from the literature indicate the great potential of PELNs in the regulation of immune functions, inflammation, microbiome, and tissue regeneration. Moreover, PELNs can also be used as drug carriers to enhance drug stability and cellular uptake *in vivo*. Since regulation of immune function, inflammation, microbiome, and tissue regeneration are the key phenomena usually targeted during periodontitis treatment, the PELNs hold the promising potential for periodontitis treatment. This review summarizes the recent advances in PELNs-related research that are related to the treatment of periodontitis and regeneration of periodontitis-destructed tissues and the underlying mechanisms. We also discuss the existing challenges and prospects of the application of PELNs-based therapeutic approaches for periodontitis treatment.

## Introduction

Periodontitis is a chronic inflammatory disease of periodontal tissue with a prevalence rate 50% (1). Periodontitis leads to chronic pain, gingival swelling, destruction of periodontal ligaments, and loss of alveolar bone and teeth (1). In 2017, severe periodontitis was the sixth most prevalent disease affecting 9.8% (about 796 million) global adult population (2, 3). Periodontitis not only affects oral health but also is linked to various systemic diseases including cardiovascular disease, Alzheimer’s disease, type 2 diabetes mellitus, respiratory tract infection, rheumatoid arthritis, nonalcoholic fatty liver diseases, and certain cancers (4, 5). Therefore, the effective treatment of periodontitis is vital for a healthy life. Conventional treatment approaches to periodontitis including non-surgical, surgical, and adjunctive pharmacological therapy have limitations, such as residual bacterial and calculus in the deep periodontal pocket, limited effect on inflammation regulation, limited periodontal tissue regeneration, and lack of consideration of the effect of systemic diseases (6–8).

In recent years, natural products have attracted more and more attention in the treatment of human diseases (9). It has been widely demonstrated that natural products possess anti-microbial, anti-oxidant, and anti-inflammatory properties and are widely used in the treatment of various diseases including cancer, malaria, and periodontitis (10, 11). In addition, observational studies had shown correlation between the intake of fruits and vegetables with oral health (12). Moreover, Kharaeva et al. indicated that toothpaste containing plant-derived ingredients has an additional therapeutic effect in the treatment and prevention of gingivitis and periodontitis (13). However, shortcomings of natural products such as uncertain stability, limited target specificity, and difficulty in purification limit their clinical applications. Halperin et al. first discovered evidence of the existence of PELNs in carrot cell cultures in 1967 (14). PELNs (50-500 nm in diameter) contain mRNAs, microRNAs (miRNAs), bioactive lipids, and proteins (15, 16). Compared to artificial nanocarriers, PELNs do not exert cytotoxicity on human cells (15). PELNs lipid bilayer contains high contents of glycolipids and phospholipids but lacks cholesterol, which indicates the potential application of PELNs as a tissue targeting drug carrier (16). Recent reports from literature had shown the potential of PELNs to treat various diseases, including inflammatory bowel diseases, lung inflammation, and periodontitis (17–19). The nanoparticle size, lipid membrane, specific targeting, and cargo-carrying capacity of PELNs offer better stability and fewer side effects. Ginger-derived exosome-like nanoparticles (GELNs) had shown an anti-bacterial effect on *Porphyromonas gingivalis (P. gingivalis)* (19). Although *in vitro* and animal studies had shown the therapeutic potential of PELNs to treat periodontitis, their clinical application has not been reported yet. This review summarizes the recent research advances in the PELNs related to immune-regulation and periodontitis treatment. We also discuss the shortcomings and prospects of PELNs-based immune-regulation and periodontitis treatment.

## PELNs

Extracellular vesicles are a subcellular structure of phospholipid bilayers membrane-enclosed vesicles and contain various cargos, including miRNA, mRNA, DNA, proteins, etc. Both prokaryotic and eukaryotic cells release extracellular vesicles. Extracellular vesicles fall into two broad categories: ectosomes (size: 100 to 500 nm) and exosomes (size: 30 to 150 nm) (20–23). PELNs are a kind of extracellular vesicles ranging in size from 50 to 500 nm derived from plants (15). Besides animal vesicles, PELNs have a complex content of small RNAs, proteins, lipids, and other metabolites. PELNs from various plants and fruits such as ginger, blueberry, and coconut have shown anti-inflammatory properties (24).

Compared to mammalian exosomes, PELNs have unique advantages including undetected by the immune system, higher bioavailability, and innocuity (25). PELNs were observed almost six decades ago but less attention was paid to this field (26). PELNs exhibit better bioavailability compared to miRNAs that are free or associated with proteins (27). PELNs have proven stability in the gastrointestinal tract, and several studies have demonstrated that PELNs can be used for therapeutic application by oral or intranasal administration (28–30). Compared to natural products, PELNs can target specific organs and have higher solubility, higher permeation through barriers, quicker dissolution in blood, and fewer side effects (29). Reports from the literature indicate that plant-derived exosomes have potential application in the treatment of periodontitis through inflammation inhibition or periodontal pathogen inhibition (19, 28). Due to these properties, PELNs show the application prospect for the treatment and prevention of various inflammatory diseases including periodontitis.

### Composition of PELNs

**Figure 1:**
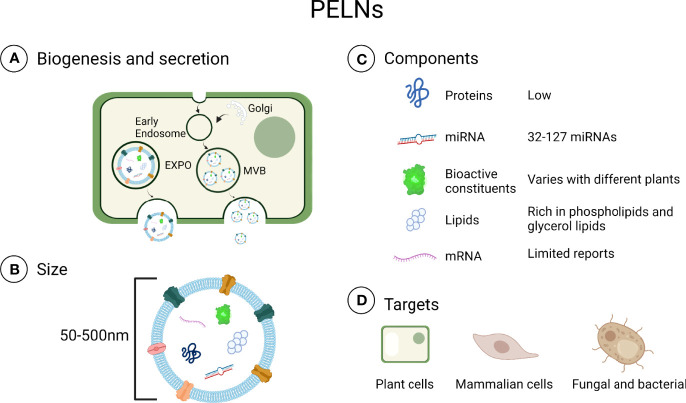
Contents in PELNs. **(A)** Biogenesis and secretion: Plant cells can secrete PELNs *via* multivesicular bodies (MVB) and exocyst positive organelles (EXPO) (43, 44). **(B)** Size: PELNs range in size from 50 to 500 nm. **(C)** Components: Generally, PELNs contain fewer proteins and miRNAs than exosomes. **(D)** Targets: PELNs can be internalized in plant cells, mammalian cells, fungi, and bacteria (28, 45–47). Created with BioRender.com.

PELNs contain various components including proteins, lipids, miRNA, and other active components such as vitamin C (31). Exosomes are derived from animal cells and are typically rich in cholesterol and sphingomyelin but PELNs are rich in phospholipids, including phosphatidic acids, phosphatidylethanolamines, and typical plant lipids (30, 32). Lipids play an important role not only in maintaining the structural stability of exosomes but also in intercellular communication (15). Among these lipids, phosphatidic acids in PELNs can inhibit *P. gingivalis* growth (19). Proteins are important components of both PELNs and mammalian exosomes, but the levels of proteins in PELNs are lower and the compositions are different (32). In mammalian exosomes, CD9 promotes cell delivery of therapeutic agents through fusing exosomal membranes to target cell membranes and CD47 could escape phagocytosis by releasing special signals and enhancing homogenous endocytosis (33). There are various proteins in PELNs, such as actin, proteolysis, aquaporin, and chloride channels proteins, which are mainly categorized into transmembrane proteins and other plasmalemma-associated proteins (25, 34). Defense proteins in some PELNs such as sunflower seeds can modulate microbiota by affecting fungal growth (35). MicroRNAs are a class of small (17-24 nucleotides) and noncoding RNAs with abilities to inhibit mRNA translation (36, 37). The latest evidence shows that plant miRNA can be absorbed in the intestine and secreted into the circulatory system (38). The previous report had shown that PELNs deliver miRNA to animals, target mammalian mRNA, and have the potential to mediate a specific tissue response (24, 39). In addition, miRNA in PELNs can be taken up by bacteria and alter microbiome composition and host physiology (28, 38, 40). But the functions and mechanisms of miRNAs in PELNs are still unknown. It is unclear how plant-based xenomiRNAs regulate gene expression in humans (41). Besides, some bioactive components such as vitamin C, citrate, 6-gingerol, and 6-shogaol have been found in PELNs (34, 42). Only a handful of studies about the biological function of bioactive components in PELNs had been reported so far. The contents of PELNs are shown in **Figure 1**.

### PELNs Biogenesis and Isolation

The extracellular vesicle formation and secretion require a multi-step cellular process that is well documented in animals. Extracellular vesicles are formed in intracavitary vesicles (ILVs) and multivesicular bodies (MVB). ILVs released into the extracellular space after fusion with the plasma membrane are exosomes (48, 49). However, the process of PELNs release from cell walls is still unknown. PELNs participate in plant-microbe interactions by safely transporting functional molecules including proteins and RNAs (50). The MVB pathway is a key process of PELNs formation (44). The endosomal sorting complex required for transport (ESCRT) binds and sequesters ubiquitinated proteins and sorts these into the ILVs of PELNs (51). However, the ESCRT genes responsible for PELNs have not yet been elucidated. Even though PELNs are secreted by most plant cells, the process of extracellular vesicles passing through the apoplastic space or cell wall is still unclear (52). In addition, exocyst-positive organelle (EXPO), in plant cells, also can expulse PELNs into the apoplast through the fusion of the outer membrane of EXPO with the plasma membrane, but the biological significance of EXPO-mediated PELNs secretion in plants is still undetermined (43).

**Figure 2:**
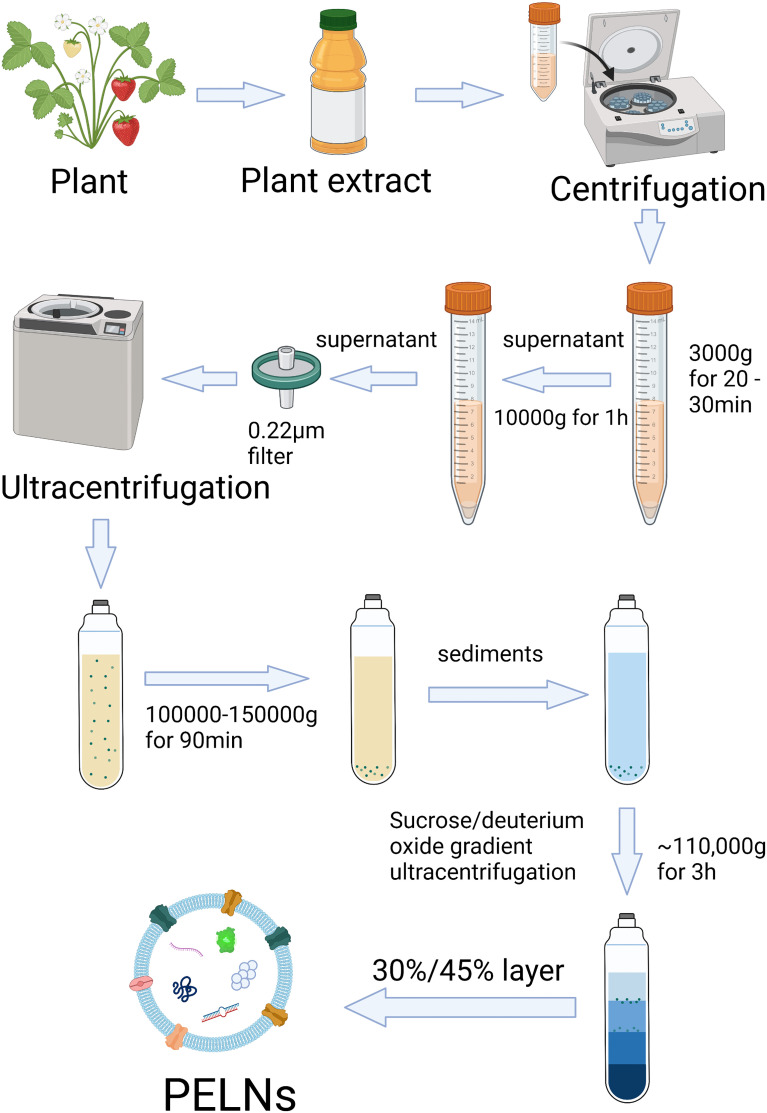
Scheme of isolation and purification of PELNs by differential ultracentrifugation and sucrose gradient ultracentrifugation. Created with BioRender.com.

The isolation of PELNs is mainly based on differential centrifugation. PELNs can be extracted from fruits, roots, stems, and leaves (24, 30, 53). The conventional method is to grind the plants into juice and then strain the juice with a colander. The collected juice goes through differential centrifugation at 3000× g (for 20-30 min) and 10,000× g (for 1 h) (24, 31). This supernatant is then subjected to centrifugation at high speed (100,000–150,000 × g) (29). Because the PELNs yield is usually contaminated by nucleic acids and protein agglomerates after differential ultracentrifugation, sucrose/deuterium oxide gradient ultracentrifugation at ~110,000 × g for 3 h at 4°C is needed for further purification (**Figure 2**) (25, 54). However, differential centrifugation also has a lot of disadvantages, e.g., low PELNs yields because a large number of nanovesicles are lost during centrifugation, retention of protein aggregates, and disruption of nanovesicles due to high centrifugal forces (55).

Polyethylene glycol (PEG)-based precipitation method is another method for PELNs isolation (56). The PEG method is a cost-effective method of PELNs isolation with comparable efficiency to differential ultracentrifugation (57). PEG methods are related to pH. Suresh et al. reported a higher yield of PELNs when PEG precipitation was carried out in pH 4 and 5 (58). Differential ultracentrifugation, PEG, density-gradient ultracentrifugation, gel filtration chromatography, ultrafiltration, immunoaffinity separation, etc. are the methods of PELNs isolation (59). Differential ultracentrifugation is still the “gold standard” due to its wide applicability, large capacity, easy scale-up, and relatively high purity (59). **Figure 2** demonstrates the procedure of the commonly used differential ultracentrifugation method of PELNs isolation.

## Internalization of PELNs by Mammalian Cells and Mechanisms of Cargo Release

Several studies had reported that PELNs can be internalized by mammalian cells and do not exert cytotoxicity (30, 31, 60). Ju et al. indicated that intestinal stem cells take up grape-derived exosome-like nanoparticles (GrELNs) through micropinocytosis, which can be inhibited by cytochalasin D (30). But the molecular mechanism of micropinocytosis of PELNs in mammalian cells is still unclear. Based on the similarity of components and structure between PELNs and exosomes, it is reasonable to guess that PELNs can be internalized by way of fusion and endocytosis just like mammalian cell exosomes (61, 62). Exosomal membrane proteins play an important role in the uptake of exosomes (61). So further study and analysis of membrane proteins of PELNs are the keys to understanding the specific mechanism of internalization of PELNs in mammalian cells. Besides, mammalian exosomes can induce cellular responses through membrane-bound or soluble signaling, which does not require internalization (63). Whether PELNs can induce cellular responses in a similar way to mammalian exosomes still needs to be further investigated.

**Figure 3:**
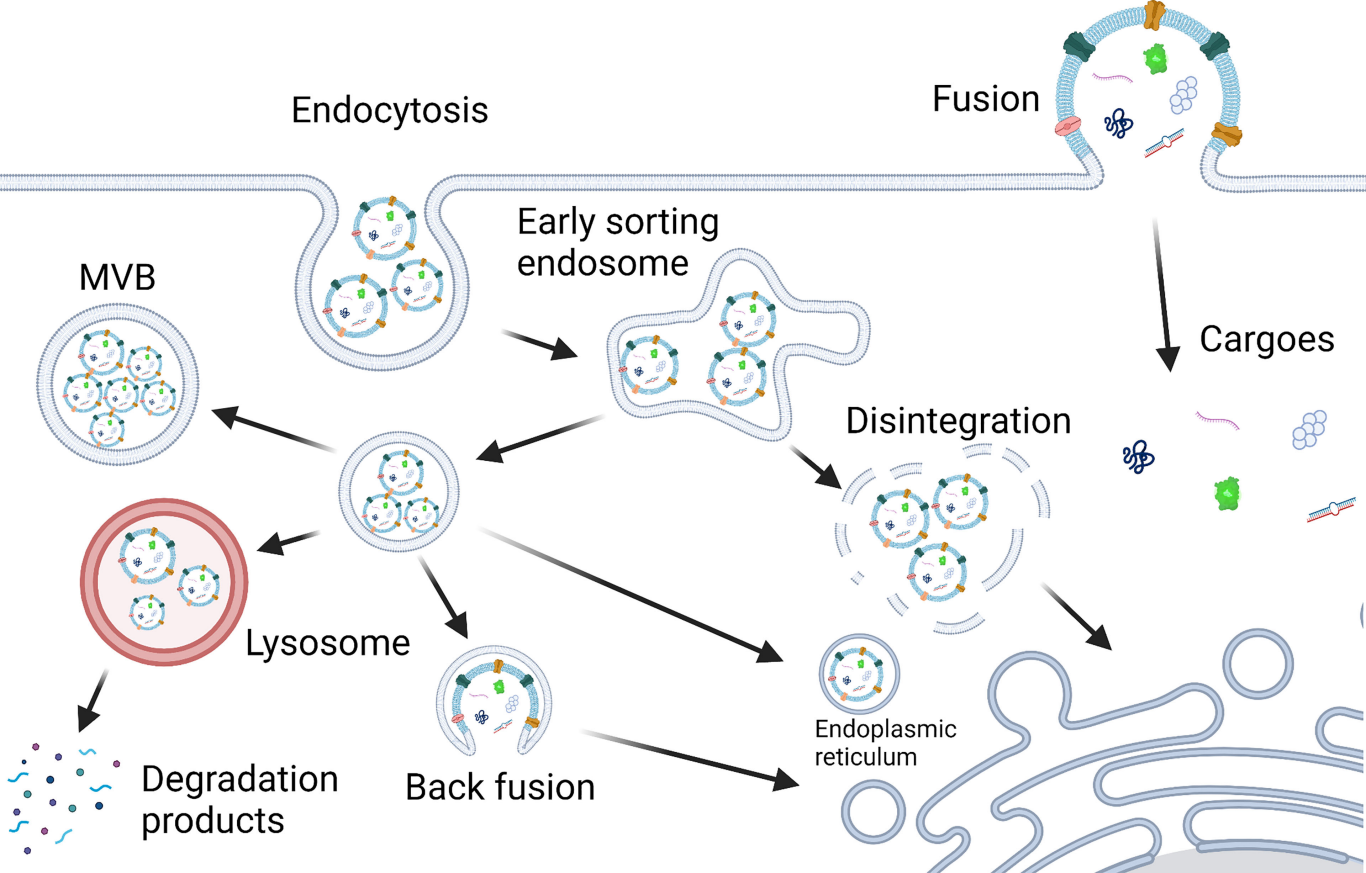
Scheme of PELNs internalization in mammalian cells and cargo release. Mammalian cells take up PELNs possibly through endocytosis and fusion. Cargoes in PELNs can be released into the cytoplasm in different ways, i.e., fusion with the lysosome, the disintegration of the early sorting endosome, and fusion with the endoplasmic reticulum, endosomal membrane and plasma membrane (22, 64). Created with BioRender.com.

The PELNs taken up by cells through fusion can release cargoes into the cytoplasm directly. But the fate of PELNs taken up through endocytosis may be different. Typically, PELNs taken up by cells may transport to the lysosome and lead to degradation-based cargo release (62). Under certain circumstances, cargoes in PELNs can be released into the cytoplasm in different ways including fusion with the lysosome, the disintegration of the early sorting endosome, and fusion with the endoplasmic reticulum and endosomal membrane (64). The possible mechanisms of PELNs internalization in mammalian cells and cargo release are depicted in **Figure 3**.

## PELNs-Mediated Regulation of Human Cells’ Functions

Recently, cross-kingdom regulation of human transcripts by plant miRNAs has been demonstrated (24). PELNs have shown anti-inflammatory properties in human cells. According to a recent report, GELNs show anti-inflammation effects by inhibiting the expression of Nsp12 in lung epithelial cell-exosome-mediated inhibition of macrophagic inflammation (18). Teng et al. reported that GELN RNAs ameliorate mouse colitis by inducing gut probiotic Lactobacillus rhamnosus GG (LGG) indole-3-carboxaldehyde, which promotes the expression of interleukin (IL)-22 in gut lymphocytes of mice through activation of the aryl hydrocarbon receptor (AHR) signaling pathway (28). Exosome-like nanoparticles (ELNs) from blueberry counter the response to tumor necrosis factor (TNF-ɑ)-induced change in gene expression in EA. hy926 cells, pretreatment with blueberry-derived ELNs counters TNF-ɑ-induced reactive oxygen species generation and loss of cell viability and modulates the differential expression of 29 genes induced by TNF-ɑ compared to control (60).

In addition to anti-inflammatory properties, PELNs have shown anti-tumorigenic properties. Berry anthocyanidins-derived ELNs inhibit ovarian cancer cell proliferation *in vitro* and tumor growth *in vivo* (65). Citrus-limon juice-derived ELNs inhibit tumor cell growth through a significant downregulation of the Acetyl-CoA Carboxylase 1 (ACACA) (66). Engineered ELNs from Asparagus cochinchinensis show antitumor activity *via* inducing apoptotic pathways (67).

## Treatment of Periodontitis

Biological activities targeted during periodontitis prevention and treatment and periodontal tissue regeneration include inflammation regulation, anti-bacterial, immune-regulation, osteogenesis, periodontal ligament regeneration, and angiogenesis. Scaling and root planing (SRP), the gold standard method for dental plaque removal, has a significant anti-bacterial effect and inhibits the development of periodontal diseases (6, 68). But the complex root anatomy and recolonization of microbiota limits the efficacy of SRP (6–8). Regenerative surgeries have been used in promoting osteogenesis and periodontal ligament regeneration, but the indication and efficacy are limited (69, 70). Although various materials such as autogenous bone, allogeneic bone, and alloplastic substitutes, are used in regenerative surgery, limited materials are currently available with the true periodontal regeneration potential (71, 72).

To overcome the disadvantages of existing therapeutic approaches, adjunctive pharmacological therapies have been used in periodontitis treatment. Antibiotics brought additional benefits as an adjunct in periodontitis treatment, but the risks such as bacterial resistance, hypersensitivity, and superinfection limit the clinical application (73–75). Natural products attract more and more attention in periodontitis treatment due to their therapeutic potential, cost-effectiveness, enough source, and safety. A series of natural products such as honey, propolis, cannabidiol, and green tea show various benefits in periodontitis treatment and prevention including inhibition of periodontal pathogens, anti-inflammation, immunomodulation, and osteogenesis (76–85). But the low stability, uncertain bioavailability, and limited therapeutic effect of natural products limit their clinical application.

## The Possible Role of PELNs in the Prevention and Treatment of Periodontitis

PELNs have shown anti-inflammatory, microbiome modulatory, immunomodulatory, and tissue regenerative properties that could be beneficial for the prevention and treatment of periodontitis. To achieve superior therapeutic effects against periodontitis, PELNs can be used in drug delivery systems to increase the bioavailability and biodistribution of the drugs (25). Compared with natural products, PELNs have a broader application range and higher stability. Unlike mammalian exosomes, PELNs can be easily isolated and purified in large quantities, and have better biocompatibility (18). Since PELNs come from plants, there are fewer ethical issues during clinical applications. Therefore, with these properties and advantages, PELNs have shown great value in the prevention and treatment of periodontitis (**Table 1**).

**Table 1:** Overview of PELNs biological activities that could be applied in periodontitis treatment.

PELNs	Function	Mechanism	Ref.
Grapefruit	Immune-regulation and anti-inflammation	Increase nuclear translocation of Nrf2 in macrophages	(86)
Ginger		Reduce TNF-α, IL-6, and IL-1β, inhibit NLRP3, and increase IL-10 and IL-22 in colitis mice	(34, 53)
Cabbage		Reduce IL-1β, IL-6, and COX-2 in macrophages	(55)
Red cabbage		Reduce IL-1β and IL-6 in macrophages	(55)
Carrot		Increase nuclear translocation of Nrf2 in macrophages	(86)
Blueberry		Reverse the effect of IL-6, IL1RL1, MAPK1, ICAM1, TRL8, and TNF-α in endothelial cells.Decrease the level of reactive oxygen species and Bax protein, and induce the expression of Bcl-2 and HO-1 in human hepatocytes	(60, 87)
Strawberry		Deliver vitamin C to adipose-derived mesenchymal stem cells	(31)
Nut		Reduce Tnfrsf1a protein and dampen the TNF-α signaling pathway in adipocytes	(88)
Ginseng		Polarize M1 macrophages and repress M2 macrophages	(89)
Garlic		Inhibit NLRP3 inflammasome activation in macrophages	(90)
Orange		Modulate the expression of HMOX-1, ICAM1, OCLN, CLDN1, and MLCK in intestinal epithelial cells	(91)
Tea		Inhibit the expression of TNF-α, IL-6, and IL-12, increase HO-1 expression level, and eliminate reactive oxygen species in macrophages	(92)
Mulberry bark		HSPA8 activates the AhR signaling pathway and induces the production of anti-microbial peptides in mice	(17)
Ginger	Modulate microbiota	Reduce FimA expression in *P. gingivalis* to inhibit its’ adhesion to epithelial cells	(19)
Lemon		Induce tRNA decay in LGG and treat *Clostridioides difficile* infection by enhancing the survivability of probiotics	(93, 94)
Tea		Increase overall abundance and diversity of gut microbiota	(92)
Grape	Regeneration	Induce proliferation of Lgr5hi intestinal stem cells	(30)
Wheat		Enhance mRNA level of collagen type I and promote proliferation and migration of endothelial, epithelial, and dermal fibroblast cells	(95)
Green tea		Inhibit the expression of MMP12, MMP13, and NOTCH3, and increase FGF12 in keratinocytes	(96)
Ginseng		Inhibit the expression of MMP13 and NOTCH3 in keratinocytes	(96)

### Immune Regulation and Anti-Inflammation

Macrophages are important parts of the immune system. In periodontitis, macrophages mediate the development and progression of periodontitis through M1 and M2 polarization (97). M1 macrophages produce a series of pro-inflammatory factors including TNF-α and IL-6 to kill bacteria, promote inflammation, and activate osteoclasts that cause absorption of the alveolar ridge. In contrast, M2 macrophages produce anti-inflammatory factors including IL-10 and transforming growth factor (TGF)-β to exert anti-inflammation and angiogenic effects, and activate osteoblasts to restore bone tissue (97–99). A recent study indicated that PELNs can be absorbed by intestinal macrophages and regulate immune response (86). GELNs can be absorbed by macrophages and upregulate the expression of heme oxygenase-1 (HO-1), IL-6, and IL-10. Carrot-derived ELNs induce IL-10 expression in macrophages. Grapefruit, carrot, and ginger-derived ELNs promote activation of nuclear factor (erythroid-derived 2)-like-2 (Nrf2) in macrophages (86). GELNs block the assembly of the NLRP3 inflammasome in macrophages (53). In addition, ginseng-derived ELNs suppress IL-4 and IL-13-induced M2-like polarization of macrophages and increased the secretion of M1-macrophage-associated cytokines including TNF-α, IL-12, and IL-6 (89).

Shreds of literature have reported that ginger, grapes, grapefruit, carrots, and blueberry-derived ELNs have anti-inflammatory effects (18, 60, 86, 100). Mu et al. demonstrated that GELNs induce the expression of heme oxygenase-1 and IL-10 in macrophages, and grapefruit, ginger, and carrot-derived ELNs promote activation of Nrf2 in macrophages (86). GrELNs cause significant induction of intestinal stem cells through the Wnt/β-catenin pathway, which protects mice from dextran sulfate sodium-induced colitis (30). Cabbage and red cabbage-derived ELNs decrease the levels of IL-6, IL-1β, and COX-2 expression in LPS-treated macrophages, showing clear anti-inflammatory effects (55). Orange (*Citrus sinensis*)-derived ELNs limit inflammation and restore the functional intestinal barrier by altering the expression of HMOX-1, ICAM1, OCLN, CLDN1, and MLCK (91). The anti-inflammatory properties of PELNs could be possibly attributed to the micro-RNA in PELNs. Aquilano et al. reported that mimics of miR159a and miR156c target Tnfrsf1a gene transcript in adipocytes. Nuts (*Juglans california, Corylus avellana, and Juglans regia*)-derived ELNs containing miR159a and miR156c show anti-inflammatory effects through reducing Tnfrsf1a protein and dampening TNF-α signaling in adipocytes (88). Aly-miR396a-5p present in GELNs inhibits inflammation and cell apoptosis by inhibiting the expression of the Nsp12 viral gene (18). Besides, the natural substance phenolic compounds may also be responsible for the anti-inflammatory effects of PELNs. GrELNs loaded with phenolic compounds inhibit colitis inflammation by decreasing TNF-α and NF-κB (100). Lipids of PELNs also show anti-inflammatory properties. Lipids in Broccoli-derived ELNs inhibit gut inflammation by driving the induction of CD11c tolerogenic dendritic cells (101). A recent report indicated that proteins in PELNs also show anti-inflammatory properties. Mulberry bark-derived ELNs prevent mouse colitis through Mulberry bark ELNs-derived heat stock protein HSP8 induced AhR/COPS8 pathway (17). The phospholipid-based vesicular system enhances the delivery and safety of aceclofenac by topical route (102). As nanoparticles with bilayer phospholipid structure, PELNs can be used as a carrier for anti-inflammatory drug loading. Orally administrated infliximab-loaded GELNs show gastrointestinal stability, colon-targeted delivery, high intestinal epithelium permeability, and better efficacy in colitis than the intravenously administered infliximab (103). PELNs are innocuous and non-immunogenic nanoparticles with higher uptake in human cells compared with other artificial nanoparticles used in drug delivery (15). In addition, natural active components in PELNs could exert a better therapeutic effect than artificially synthesized nanoparticles such as liposomes. Although this field of research has not been explored in-depth, the reports from literature suggest the important role of PELNs in the treatment of inflammatory diseases. Inflammation plays an important role in the tissue destruction of periodontitis. Therefore, PELNs could have the potential to decrease periodontal tissue destruction and slow the rate periodontitis progression through inhibition of inflammation.

### Modulation of Microbiota Composition

As a secondary inflammatory response caused by oral microbiome dysbiosis, periodontitis is initiated by the host immune response to changes in the oral microbiome (104). Among the more than 500 bacterial species living in the oral cavity, a bacterial complex called “red complex”, which is made of *P. gingivalis*,* Treponema denticola*, and* Tannerella forsythia*, express various virulence factors, which enable these bacteria to colonize in subgingival space, destroy the host’s defense system, invade periodontal tissue, and promote the host’s immune destruction response (105). Given the role of microbiota in periodontitis, it is crucial to maintain the dynamic equilibrium of oral microecology (106). It is widely accepted that PELNs have a key role in plant-pathogen interactions (107). PELNs have shown regulatory effects not only in immune cells but also in microbiota. PELNs play an important role in immune responses against the fungal pathogen in plants. For instance, Arabidopsis-derived ELNs delivered host sRNA into pathogenic B. cinerea to inhibit its pathogenicity (108). PELNs also inhibit various pathogens such as *P. gingivalis* and promote the growth of probiotics. Sundaram et al. reported that GELNs inhibit *P. gingivalis* growth through phosphatidic acid by binding to hemin-binding protein 35 (HBP35) on the surface of *P. gingivalis (* 19). Besides, PELNs show microbiome regulatory properties through the promotion of the growth of probiotics. Lemon-derived ELNs protect LGG from bile possibly through inhibition of the expression of Msps protein, induction of specific bacterial tRNA decay, and inhibition of S.24-7 growth (93). Lemon-derived ELNs manipulate LGG and Streptococcus thermophilus ST-21 to protect mice from Clostridioides difficile infection (94). In addition, plant miRNAs in PELNs modulate microbiota gene expression contributing to the dietary effect on the gut microbiota community’s assembly (109). PELNs-derived small RNAs shape the homeostatic balance between host immunity and gut microbiota and regulate microbiota (28). Teng et al. indicated that GELNs-RNA induces Lactobacillus rhamnosus indole-3-carboxaldehyde to promote the expression of IL-22 through activation of the AHR signaling pathway exerting anti-microbiota immunity and tissue repair (28). With the ability to modulate oral and gut microbiota, it is reasonable to suppose that PELNs have the potential to be used in the treatment of periodontitis through the regulation of the periodontal microbiome.

### Periodontal Tissue Regeneration

**Figure 4:**
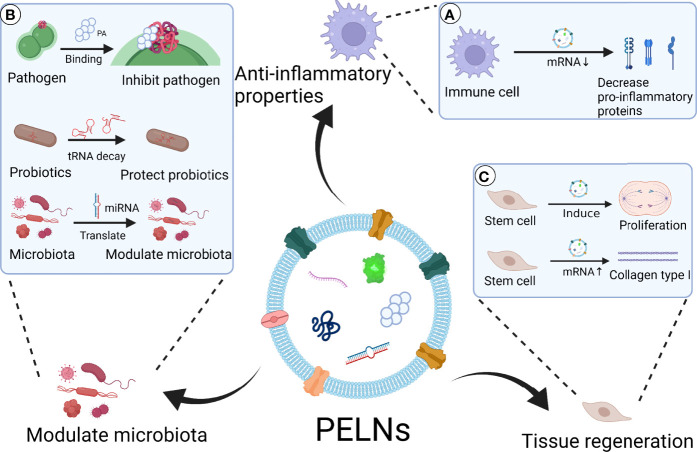
Scheme of PELNs’ possible application in the treatment of periodontitis. PELNs show therapeutic potential for periodontitis *via* anti-inflammatory effect, microbiota modulation, and tissue regeneration. **(A)** Anti-inflammatory properties: PELNs inhibit pro-inflammatory protein expression in macrophages, such as IL-1β, IL-6, and TNF-α (55). **(B)** Modulate microbiota: PELNs inhibit pathogenic bacteria such as *P. gingivalis* through PA binding to HBP35 (19). PELNs protect probiotics through decay tRNA in probiotics (93). PELNs transform miRNA to microbiota, which has the potential to modulate oral microbiota. **(C)** Tissue regeneration: PELNs promote the proliferation of stem cells through activation of Wnt signals (30, 114). PENLs promote the production of collagen type I in epithelial cells (95).Created with BioRender.com.

The progressive loss of periodontal tissues is one of the characterizations of periodontitis, and the unique anatomy and composition of periodontal tissues make periodontal tissue regeneration a complex process (110). The reconstruction of periodontal tissues including cementum, periodontal ligament fibers, and bone remains a major challenge in periodontal treatment (111). Osteogenesis, inflammatory response, angiogenesis, and remodeling play a significant role in periodontal bone regeneration (112). Reports from the literature indicate that mammalian exosomes stimulate both osteogenesis and angiogenesis (113). With component and structure similarity to mammalian exosomes, PELNs could have application potential in tissue regeneration. According to recent research, PELNs have regulatory effects on tissue regeneration. Sahin et al. reported that wheat-derived ELNs promote collagen type I production, proliferation, and migration of fibroblasts (95). Wheat-derived ELNs exert anti-apoptotic activity in human dermal fibroblast, human keratinocyte cell, and human keratinocyte cell. In addition, wheat-derived ELNs induce angiogenesis in human umbilical vein endothelial cells (95). These results suggest the possible periodontal soft tissue regeneration and angiogenesis potential of PELNs. Besides, Syrah GrELNs induce the expression of leucine-rich repeat-containing G-protein-coupled receptor 5 of intestinal stem cells through activating downstream canonical Wnt signals, which is beneficial for the regeneration of intestinal epithelium (30, 114). Lipid contents in GrELNs promote the proliferation of intestinal stem cells (30). Whether PELNs can promote periodontal tissue regeneration by inducing tissue-specific differentiation of precursor cells remains a mystery. Green tea and ginseng-derived ELNs show potential benefits to skin regeneration, through modulating the expression of genes including MMP12, MMP13, HS3ST3A1, FGF12, LOX, VIM, ELOVLs, KRT1, and NOTCH3 in keratinocytes, which are related to skin aging, regeneration, barriers, and moisturizing (96). According to the existing data, we can reasonably guess that PELNs have a potential therapeutic effect on promoting the proliferation of periodontal stem cells and promoting the repair and regeneration of periodontal tissue. The possible applications of PELNs in the treatment of periodontitis are shown in **Figure 4**.

## Summary

As a type of plant-derived extracellular nanovesicles, PELNs can be taken into the human circulation from the gut and participate in cross-kingdom communication. The biological properties of PELNs are based on the transmission of miRNAs, proteins, lipids, and other active components. Recent studies indicated that plants communicate with mammalian cells and bacteria through PELNs, and miRNAs in PELNs show the potential to regulate human mRNAs’ activities (24, 28, 86). Increasing evidence indicated that PELNs had great potential in immune regulation and treatment/prevention of periodontitis due to their drug delivery ability and various therapeutic effects including anti-inflammatory, immunomodulatory, microbiota modulatory, and regenerative effects. Besides, compared with conventional chemical drugs and natural products, the special properties including therapeutic and drug delivery ability, stability, and safety enable the PELNs-based therapeutic approaches to break the limitations of the existing periodontitis treatment. Furthermore, compared with mainstream oral hygiene maintenance methods such as chlorhexidine, PELNs are more suitable for daily prevention of periodontitis, such as mouthwash, toothpaste, and chewing gum.

## Challenges

Although PELNs show various advantages in the prevention and treatment of periodontitis, there are still some barriers that need to be overcome to explore possibilities for their clinical applications. PELNs as nanocarriers cannot load a high quantity of drugs. To augment cargo loading capability, PELNs amalgamation with artificially synthesized liposomes by the membrane fusion technique may be a feasible approach (25). In addition, the mechanism of PELNs uptake by mammalian cells is also unclear. To further explore the potential of clinical application, it is necessary to determine the mechanisms that facilitate PELNs’ internalization in mammalian cells. The standards of biochemical analysis and biomarker confirmation of PELNs need to be developed for the quality control and engineering process. PELNs derived from the same species of plant may vary from batch to batch. Therefore, it is necessary to develop strategies to minimize the batch-dependent variation of PELNs’ contents.

## Prospects

**Figure 5:**
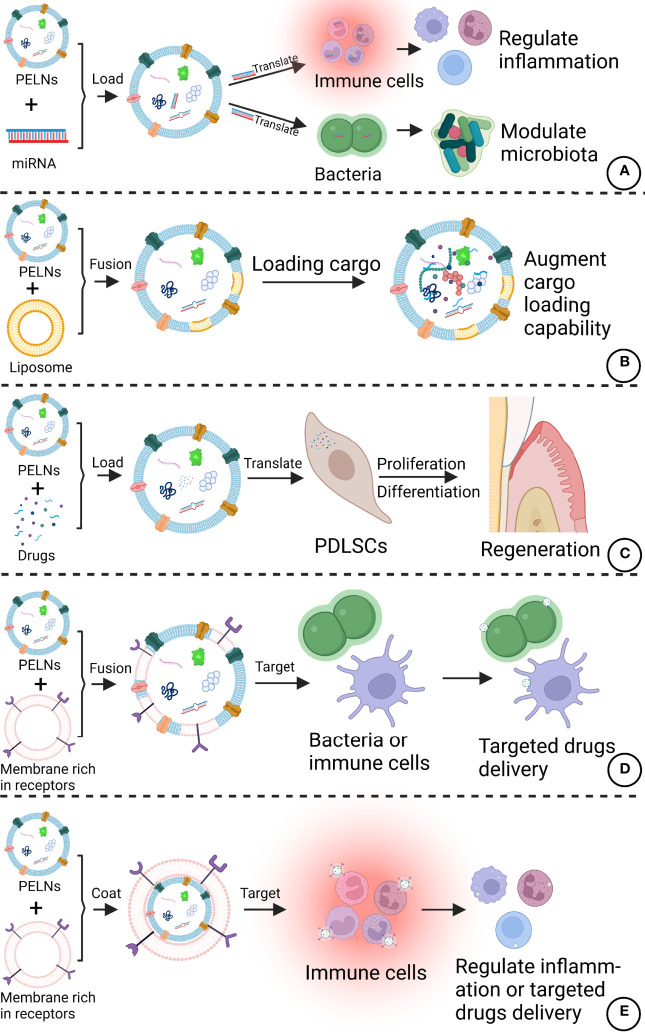
Potential modifications of PELNs that can improve therapeutic efficacy against periodontitis. **(A)** PELNs can be loaded with specific miRNAs targeting immune cells and bacteria. **(B)** The amalgamation of liposomes with PELNs could augment the cargo loading capability of PELNs. **(C)** PELNs can be loaded with the drugs of interest to promote PDLSC functions. **(D)** PELNs fusion with a membrane rich in special receptors can improve target bacteria or cells in the periodontal region. **(E)** Coating of membrane rich in special receptors in PELNs can target immune cells. Created with BioRender.com.

As a cell-free therapy, PELNs are safer than cell therapy and PELNs can target specific cells or organs (115). Compared with other cell-free therapies, such as mammal exosomes, PELNs are easier to extract and have unique advantages including biocompatibility, large-scale production capability, and low immunogenicity (116). PELNs also have the potential to use in oral care products such as buccal tablets for daily prevention of periodontitis. As lipid-based nanoparticles, PELNs can carry and deliver hydrophilic, hydrophobic, and lipophilic drugs (117). It has been reported that Acerola-derived ELNs can encapsulate nucleic acids without the use of special reagents (118, 119). Various methods such as loading by electroporation, saponin membrane permeabilization, and extrusion had been developed drug loading in exosomes, which can be adapted to load desired drugs in PELNs. Besides, PELNs have good stability and can protect the contents from physicochemical damage *in vivo*. The encapsulated siRNA in PELNs is shown to be stable against physical stimuli including sterilization, homogenization, and sonication (119). These results allow us to hypothesize that PELNs can load anti-inflammatory, anti-microbial drugs or special siRNA for periodontal treatment in the future. In addition, PELNs can be imparted with target specificity through investigating the surface tailoring of PELNs (25). It is possible to modulate the tropisms of PELNs by introducing tissue-specific peptides or proteins onto the surfaces of PELNs (120). Membrane fusion with other extracellular vesicles or cell membranes with special receptors may also be a feasible way to enhance the targeted specificity of PELNs. The combination of PELNs and new clinical techniques of minimally invasive targeted delivery such as convection-enhanced delivery may also extend the application of PELNs by improving the reliability and limiting the confusion of various target bioavailability (121). Wang et al. proposed a new method that GrELNs coated with inflammation-related receptor enriched membranes of activated leukocytes, which showed better target ability to inflammatory tumor tissues (122). Therefore, PELNs have great potential to be developed as a drug delivery system for periodontitis treatment. Potential modifications of PELNs that can improve therapeutic efficacy against periodontitis are listed in **Figure 5**.

## Conclusions

In conclusion, PELNs have shown many advantages including biocompatibility, specific cell targeting capability, cost-effectiveness, large-scale production, and drug delivery capability. The biological activities of PELNs including immune-regulation, effect on microbiome homeostasis, inflammation modulation, and tissue regeneration could be applied to the treatment of periodontitis. Although great progress has been obtained in the field of PELNs especially edible PELNs in the last decade, this field is still in its infancy. A lot of challenges should be overcome before the clinical application of PELNs. The relationship between components of natural products and PELNs has not been fully clarified. It is unclear which PELNs have therapeutic effects on periodontitis. The active components and mechanism of PELNs in the treatment of periodontitis are still unknown. In addition, although some PELNs show benefits to tissue regeneration, only one study had been reported so far regarding the therapeutic role of PELNs in periodontal disease. Based on the results from the recent literature, the direct use of PELNs to treat periodontitis or loading drugs and miRNAs, shRNAs, and siRNAs in PELNs to treat periodontitis could be a new era in the prevention and treatment of periodontitis.

## Author Contributions

LY, LW, and JP conceived the manuscript. ZZ, YY, GZ, and LZ wrote the original draft. LW, JP, SX, HC, ZO, and JC revised the manuscript. All authors contributed to the article and approved the submitted version.

## Funding

This work was supported by the National Natural Science Foundation of China (82150410451) and Guangdong Medical Research Foundation (A2019250): Long-chain noncoding RNA BMP2-1 affects human periodontal ligament stem cells by regulating BMP2.

## Conflict of Interest

The authors declare that the research was conducted in the absence of any commercial or financial relationships that could be construed as a potential conflict of interest.

## Publisher’s Note

All claims expressed in this article are solely those of the authors and do not necessarily represent those of their affiliated organizations, or those of the publisher, the editors and the reviewers. Any product that may be evaluated in this article, or claim that may be made by its manufacturer, is not guaranteed or endorsed by the publisher.
